# Evaluation of the Apple iPhone 12 Pro LiDAR for an Application in Geosciences

**DOI:** 10.1038/s41598-021-01763-9

**Published:** 2021-11-15

**Authors:** Gregor Luetzenburg, Aart Kroon, Anders A. Bjørk

**Affiliations:** Department of Geosciences and Natural Resource Management, University of Copenhagen, Copenhagen, Denmark

**Keywords:** Geomorphology, Semiconductor lasers

## Abstract

Traditionally, topographic surveying in earth sciences requires high financial investments, elaborate logistics, complicated training of staff and extensive data processing. Recently, off-the-shelf drones with optical sensors already reduced the costs for obtaining a high-resolution dataset of an Earth surface considerably. Nevertheless, costs and complexity associated with topographic surveying are still high. In 2020, Apple Inc. released the iPad Pro 2020 and the iPhone 12 Pro with novel build-in LiDAR sensors. Here we investigate the basic technical capabilities of the LiDAR sensors and we test the application at a coastal cliff in Denmark. The results are compared to state-of-the-art Structure from Motion Multi-View Stereo (SfM MVS) point clouds. The LiDAR sensors create accurate high-resolution models of small objects with a side length > 10 cm with an absolute accuracy of ± 1 cm. 3D models with the dimensions of up to 130 × 15 × 10 m of a coastal cliff with an absolute accuracy of ± 10 cm are compiled. Overall, the versatility in handling outweighs the range limitations, making the Apple LiDAR devices cost-effective alternatives to established techniques in remote sensing with possible fields of application for a wide range of geo-scientific areas and teaching.

## Introduction

In geosciences, terrestrial laser-scanning and airborne laser-scanning (TLS & ALS) techniques are applied for topographic land surveying on a wide range of scales^1–3^. LiDAR is a common technique to measure distances by timing the return pulse, emitted from a laser transmitter to the laser receiver^4^. The rapid evolution of digital processing techniques, as well as a new generation of technologies in remote sensing, is leading to a revolution in digital elevation modeling and geomorphological terrain analysis^5^. Nevertheless, the acquisition of a digital terrain model, independent from the scale, requires high capital and logistical costs in the order of several thousand of euros, especially with airborne laser-scanning techniques^6^. Therefore, data acquisition of airborne large-scale remote sensing datasets is often outsourced to third party organizations, concomitantly data acquisition is planned well in advance regardless of unforeseeable events.

However, many processes studied in geosciences, like coastal cliff erosion, are irregular processes in time and space. Coarse temporal resolution observations may limit pivotal process understanding^7^. Terrestrial laser-scanning (TLS) allows the acquisition of digital terrain models up to a medium-scale of a few kilometers with a high temporal and spatial resolution^8,9^. Usually, terrestrial laser scanners cost at least several thousands of euros, requiring trained operators and line of sight, only allowing a limited number of scanning positions, and are restricted in access to rough terrain. Hand-held mobile laser scanner (HMLS) can overcome some of those limitations, but are rarely used in geosciences^10^. Furthermore, TLS and HMLS still require highly skilled field handling and post processing^11^.

Recent advances in photogrammetry and the availability of lightweight unmanned aerial vehicles (UAVs) offer a potential low-cost alternative to ALS and TLS in order to build 3D surface models with a high temporal and spatial resolution^6,12^. However, current high-resolution 3D models based on UAV data and Structure-from-Motion Multi-View Stereo (SfM MVS) pipelines are still expensive as they rely on differential global navigation satellite systems (DGNSS), ground control points (GCPs), commercial software and data processing on an external computing device^13^. Moreover, UAV missions rely on certain weather conditions, entail a sophisticated setup, high operational complexity and SfM MVS techniques present challenges by managing and processing large volumes of data^14,15^. Furthermore, the use of UAVs are subject to a growing number of regulations and restrictions prohibiting flight. Multiple studies compared the two techniques concluding both should coexist, with their respective primary fields of application^16–18^.

The commonness of smartphones nowadays, together with advances in sensor technologies, opens new possibilities for scientific applications as well as low-cost, crowd-sourced observations in mapping surface changes and the participation of citizens in science^19–21^. SfM MVS smartphone photogrammetry takes advantage of the build in accelerometer, magnetometer, gyroscope and GNSS antennas for model scaling and registration. However, photo position and orientation cannot be used for satisfactory model registration, making an elaborate model post processing necessary, enabling only experts to use the advantages of smartphone photogrammetry^22^.

A LiDAR scanner ubiquitously available on consumer-grade devices was presented with the introduction of the iPad Pro 2020 11-inch and 12.9-inch display (hereafter iPad) on March 25, 2020 and the iPhone 12 Pro and iPhone 12 Pro Max (hereafter iPhone) on October 23, 2020 by Apple Inc.. Comparisons between the iPad LiDAR sensor, a hand-held personal laser scanning approach and traditional forest inventory equipment already demonstrated a high detection rate of tree stems above a threshold of 10 cm diameter^23,24^.

Here, we test the novel Apple LiDAR sensor at a coastal cliff site in eastern Denmark. Coastal cliffs are quickly changing dynamic environments with a high geo-hazardous potential. They are representative for numerous research areas within the geosciences^11^. The aim of this study is to test and assess the application of the LiDAR scanner in the iPhone and iPad for geoscientific research by investigating (i) the technical capabilities, including accuracy and precision of the LiDAR sensor in a controlled environment and (ii) the usability in-situ at a coastal cliff in eastern Denmark and (iii) compare the output of the iPhone LiDAR sensor with smartphone photogrammetry.

**Figure 1:**
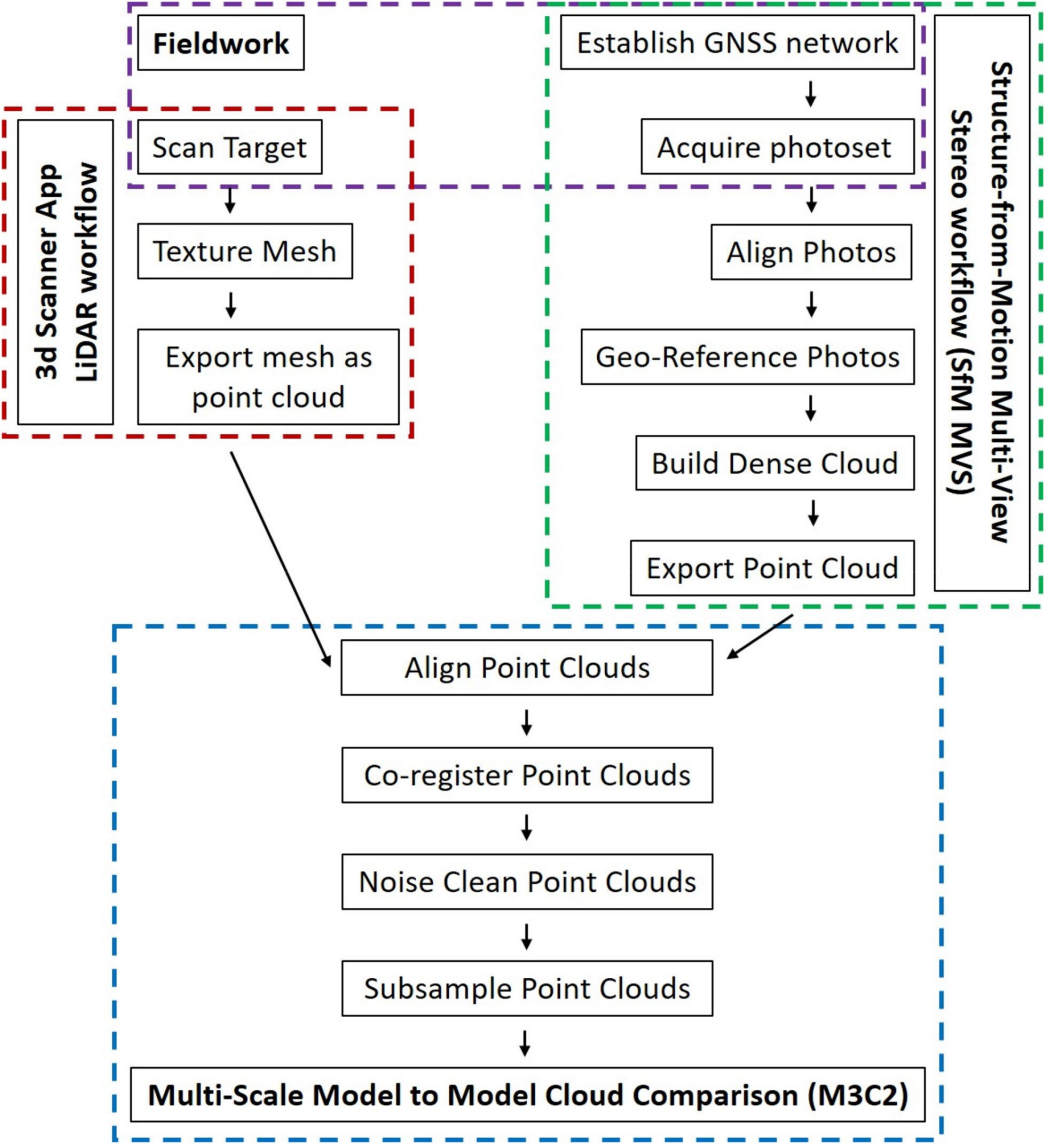
Workflow for the acquisition of the LiDAR point clouds with the ‘3d Scanner App’ (red box), the SfM MVS point clouds (green box) including fieldwork (purple box), and the Multi-Scale Model-to-Model Cloud Comparison (M3C2) in CloudCompare (blue box).

Several small objects with known dimensions were scanned to test accuracy and precision of the LiDAR sensor. Independent models of a coastal cliff were acquired with the ‘3d Scanner App’ utilizing the iPhone’s LiDAR sensor, via SfM MVS photogrammetry, and with the ‘EveryPoint’ app combining the iPhone’s LiDAR and the iPhone’s camera photos. The LiDAR models were aligned onto the SfM MVS reference models and the distances between the point clouds were analyzed (Fig. 1). GCPs were used for model alignment and fine registration was performed in CloudCompare^25^ without changing the scale of the models. In-situ model accuracy was tested by multi-scale model-to-model cloud comparison^26^ (M3C2) between the LiDAR point clouds, the SfM MVS reference point clouds and the ‘EveryPoint’ point clouds. Precision was tested by comparing several iPhone LiDAR models of the same area with each other.

The coastal cliff of Roneklint is located in eastern Denmark (55.131161 N, 12.131817 E) on the Island of Zealand facing the Baltic Sea (Supplementary Fig. S1a,b). The cliff stretches over 130 m along the shore and the average height is around 10 m. Sediments consist of sandy glacial till prone to erosion by waves. The shallow intertidal zone allows the micro-tidal wave regime to reach the cliff base during winter storm conditions that are common in the area. This makes the cliff especially vulnerable to erosion. Several deadfalls and large boulders in front of the cliff face are evidence of high erosional activity along the cliff (Supplementary Fig. S1c).

Size, topography, accessibility, and fast changing irregular processes on the cliff face are making this coastal cliff an ideal study site to test the capabilities of the iPad and iPhone LiDAR scanner in an in-situ setting.

## Results

### Technical capabilities

The laser is emitted from a Vertical Cavity Surface Emitting Laser (VCSEL) at a near infrared spectrum in a 2D array^27^. Compared to common Edge Emitting Lasers (EEL), VCSELs are convenient for mobile devices, as they can be constructed in small-dimensions featuring a feasible ratio between laser power consumption and supplied power as well as a narrow wavelength bandwidth^28^. Flash illuminating facilitates the observation of the entire field of view (FoV) at once, but it is also limiting the size and range of the FoV. The direct time of flight (dTOF) of the pulses emitted by the VCSEL is measured with a Single Photon Avalanche Photodiode (SPAD)^29^. Fabrication in Complementary Metal-Oxide-Semiconductors (CMOS) technology is leading to a cost-effective solution for SPADs^30^. Increases in power density of VCSELs in combination with SPADs makes flash-LiDAR solutions feasible for consumer-grade devices like the iPad and iPhone^31^.

**Figure 2:**
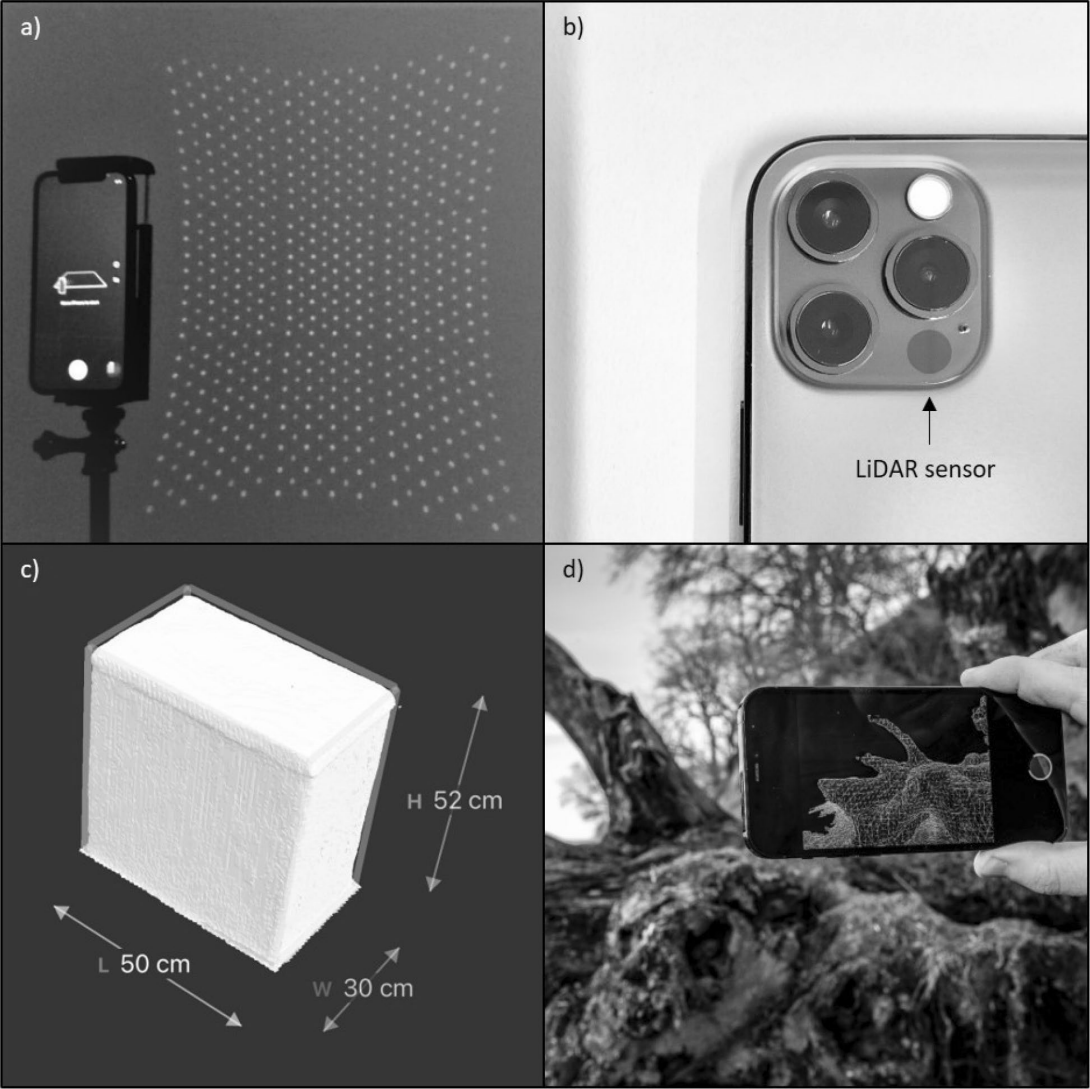
The Apple iPhone 12 Pro mounted on a selfie stick with the LiDAR sensor emitting an array of 8 × 8 points diffracted into 3 × 3 grids making a total of 576 points (**a**), Apple iPhone 12 Pro camera module (**b**), 3D model of an object with measured dimensions (**c**), 3d Scanner App scanning deadfall at Roneklint on the Apple iPhone 12 Pro (Photo credit Kent Pørksen) (**d**).

The VCSEL emits an array containing 8 × 8 points that is diffracted into 3 × 3 grids, making a total of 576 points (Fig. 2a). The focal length is equivalent to 26 mm and therefore the same as the main 12 MP camera of the iPad and iPhone (Fig. 2b). The maximum range is up to 5 m. The potential point density follows a linear trend on a logarithmic scale with 7,225 points m^−2^ at 25 cm distance and 150 points m^−2^ at 250 cm distance (Supplementary Fig. S2). No differences between the iPad and the iPhone LiDAR scanner in the total number of emitted points, point density and focal length were observed. Therefore, we conclude that there are no differences between the iPad and iPhone LiDAR sensors.

**Figure 3:**
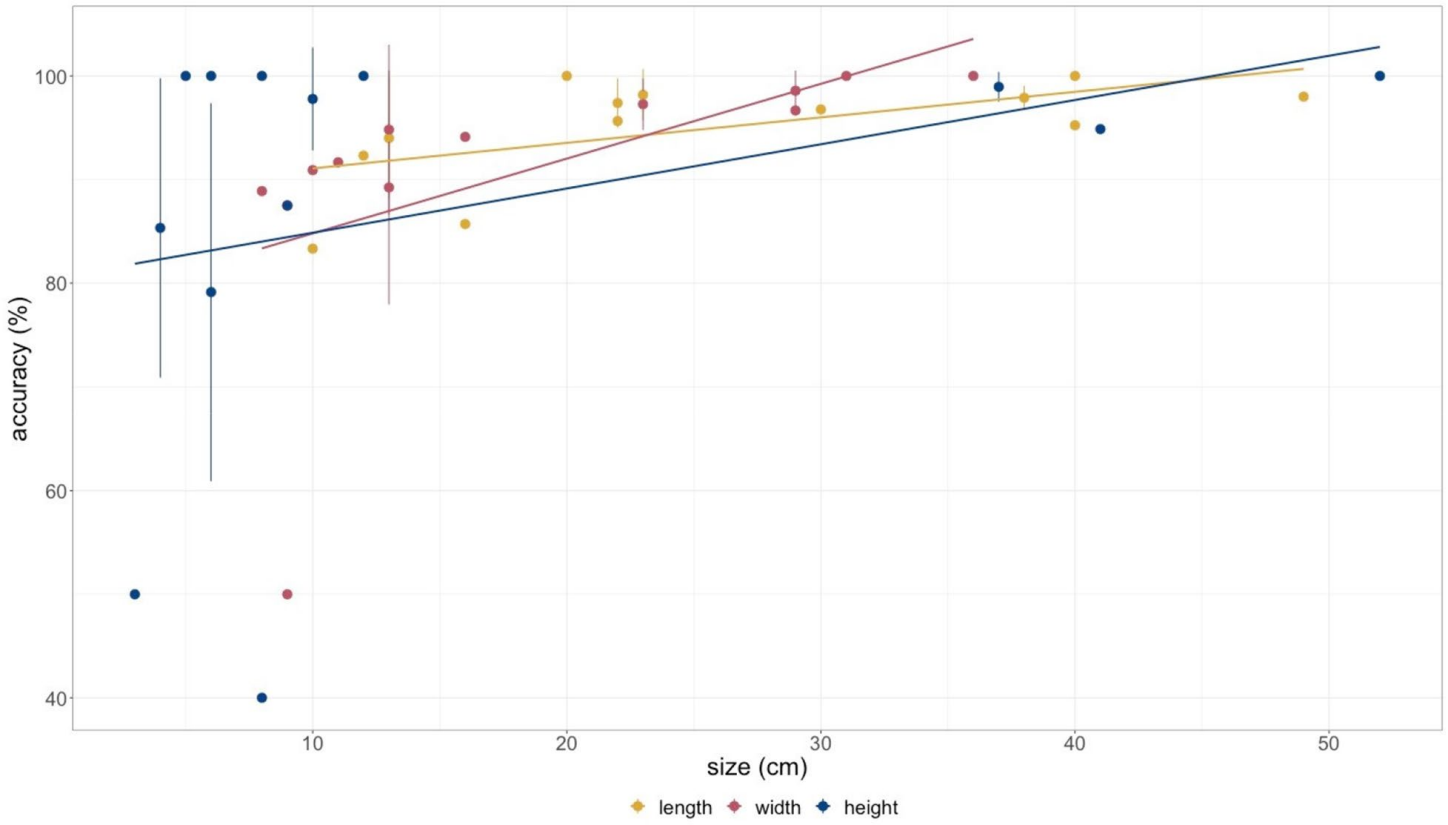
Percentage of accuracy between measured and real values of small objects for length, width and height. Lines show precision of measured values for repeated scans (n = 5) of the same object. Accuracy and precision are increasing with object size in all directions. Lines indicate linear trend lines.

Shapes of small objects are measured with an absolute accuracy of one centimeter and an error in precision of one centimeter (Fig. 2c). Precision is decreasing when scanning surfaces under 10 cm side length and the limit of detection for objects is around five cm (Fig. 3).

### 3D modeling coastal cliffs

**Figure 4:**
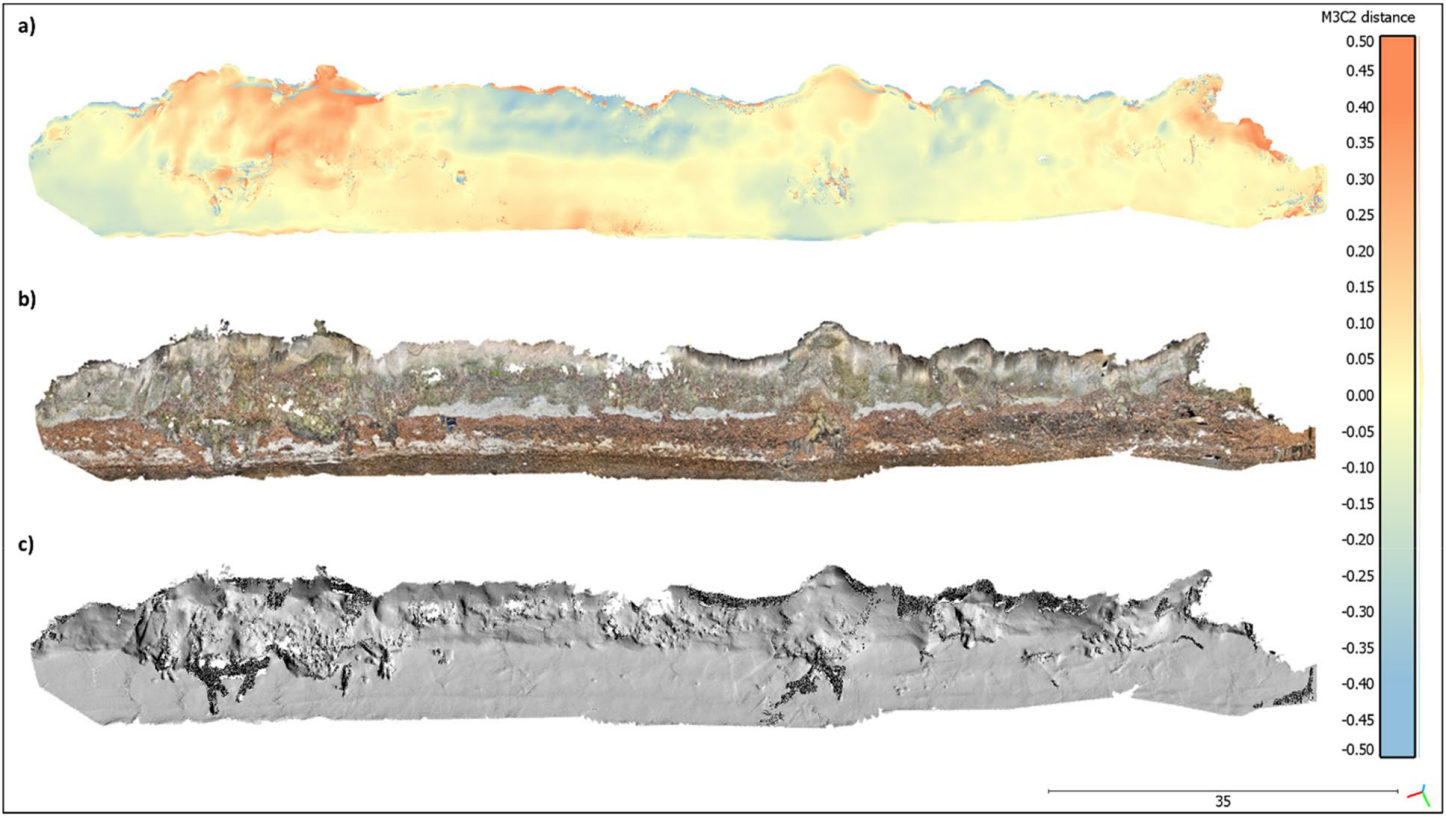
M3C2 distances in meter between SfM MVS reference point cloud and iPhone point cloud, fine registration error RMS: 0.052 m computed on 5 million points with a theoretical overlap: 75%, point clouds subsampled to 0.05 m minimal nominal spacing between points with normal directions and projection diameter calculated at 1.33 m for each point (**a**), textured iPhone LiDAR model of the cliff (**b**) iPhone LiDAR hillshade model of the cliff (**c**). Scale bar in bottom right indicates 35 m.

Scanning of the entire coastal cliff and the beach at Roneklint (length: 130 m, width: 15 m, height: 10 m) in December 2020 took about 15 min with the ‘3d Scanner App’ on the iPhone or iPad and the obtained mesh consists of around 1.5 million vertices which are textured with around 2.5 k overlapping images. Visual interpretation of the iPhone LiDAR model shows a consistent representation of the scanned surfaces (Fig. 2d). The return signal is stronger on relatively flat surfaces like the beach in front of the cliff and on un-vegetated areas on the cliff face, compared to areas covered with vegetation. Small structures such as stems and boulders are captured realistically. Texturing adds an additional layer of information to the point cloud, visualizing small shapes, ground cover characteristics and 2D objects like GCPs (Fig. 4b,c).

The average M3C2 distance between the SfM MVS based reference cloud and the LiDAR point cloud of the entire cliff is -0.11 m (std. dev. = 0.68, RMS = 0.69). M3C2 shows that for 80% of all points the maximum distance between the SfM MVS and the iPhone point cloud is smaller than 15 cm and for 92% smaller than 30 cm (Supplementary Fig. S6). Horizontal areas on the beach show smaller differences between the clouds then those on the sloped cliff face (Fig. 4a). Moreover, rough surfaces show higher differences than plane areas. As expected for a flash illuminating system, M3C2 differences are higher in areas with increased distances between laser sensor and target. Distances are smaller in areas with a higher number of GCPs, indicating a bias in distance calculation towards co-registration of the point clouds.

A total of six scans of a smaller area (length: 10 m, width: 15 m, height: 10 m), taken in December 2020 with the ‘3d Scanner App’ show a mean M3C2 distances of 0.02 m between one reference model and the remaining five models, pointing out a high precision in a real-world environment. The average M3C2 distance between the five LiDAR models and the reference LiDAR model of a small area of the cliff is smaller than 5 cm for 92% (std. dev. = 4.50) of the points (Supplementary Figs. S9, S11, S13, S15 and S17). Those results are in line with the measurement of small objects, showing that precision is decreasing for objects under 10 cm, but providing reliable results for objects above that threshold (Supplementary Figs. S8, S10, S12, S14 and S16).

To compare the iPhone LiDAR with smartphone photogrammetry, the same small area at the cliff of Roneklint was scanned again in September 2021: two times with the ‘3d Scanner App’, two photo sets were taken with the iPhone’s camera for SfM MVS models and two recordings were obtained with the ‘EveryPoint’ app (Supplementary Fig. S18a-f). Out of the six models, one SfM MVS model was selected as a reference for M3C2 comparison to the remaining five models. The beach in front of the cliff was covered in dense vegetation. Both the SfM MVS and the ‘EveryPoint’ algorithm could not create points in the area with the vegetation. Therefore, the comparison focused on the bare soil at the cliff face. Since the areas closest to the iPhone are not considered in the distance calculation, the ‘EveryPoint’ point cloud in this investigation is entirely based on photos and not a combination of iPhone LiDAR and photos. The mean M3C2 distance between the two SfM MVS point clouds are the smallest and the distances between the ‘EveryPoint’ point clouds to the reference cloud are slightly larger than those between the ‘3d Scanner App’ point clouds and the reference cloud (Supplementary Table S3). Nevertheless, the standard deviation is slightly higher for the ‘3d Scanner App’ point clouds, compared to the ‘EveryPoint’ point clouds. The main difference between the point clouds however, is the areas that are not regarded in the photogrammetry point clouds. Whereas the iPhone camera SfM MVS and the ‘EveryPoint’ photogrammetry approach give reliable results for objects that are rectangular to the recording device, the LiDAR sensor also creates point clouds for areas that are oblique to the sensor.

## Discussion

The new Apple iPad and iPhone Pro line devices have their primary field of application in small to medium scale rapid changing morphological features, ranging from centimeters up to several hundreds of meters in many different disciplines of the geosciences and beyond, e.g. Geomorphology, Geology, Forestry, Archeology. Sensor performance is equal on the iPad and the iPhone. However, the smaller size of the iPhone provides a higher versatility. Mounting the iPhone on a 1.5 m long selfie stick increases the extend of the model considerably, especially vertically along the cliff face. Nevertheless, both methods, SfM MVS and LiDAR, are only representations of the real surface along the cliff. A comparison of those methods is therefore only relative. The results of the M3C2 distance calculation of the entire cliff of Roneklint show that most values are within the RMS error of the fine registration of the models. The measured distances might therefore come from inaccuracies of the iPhone’s inertial measurement unit (IMU) and not incorrect distance measurements of the LiDAR scanner. The LiDAR technology is measuring the distance between sensor and target directly at all means, whereas SfM MVS is calculating the distance indirectly based on different perspectives.

The advantage of creating a 3D model of the close-range surroundings without any previous preparation enables straightforward and timely data collection. The common availability, convenient handling and time efficient application further empowers authorities, communities and citizen science programs to monitor environmental change with unprecedented ease. Live visualization of the captured surfaces as well as integrated data processing reduces the required hard- and software making it more cost-effective then TLS or SfM MVS techniques. Export of point clouds or meshes in common formats enables sharing data across different platforms and infrastructures. Co-registration of the automatically scaled models can be performed without global referencing, by point-pair-picking between models.

However, the limitation in range reduces the field of applications to close range and small to medium scale study sites. The utilization of a selfie stick is a simple solution to overcome the limitations in range partly. Hitherto, limits in the maximum number of raw LiDAR points restricts many applications in the geosciences where raw LiDAR data is the deliverable. Apple’s ARKit internal mesh triangulation overcomes the size limitations partly, at the cost of the 3D models accuracy. Future advances in power supply for smartphones and the combination of VCSELs with SPADS and their integration in a smartphone provide the potential for improvements in range and ubiquitous availability.

UAV based SfM MVS photogrammetry has a major advantage over smartphone photogrammetry with versatile viewing angles. Therefore, the UAV SfM MVS model of the cliff of Roneklint could reproduce both the horizontal and the vertical areas of the cliff and the beach, whereas the iPhone camera SfM MVS only covered the vertical areas of the cliff face that were rectangular to the camera. With the actively sensing laser, the LiDAR can cover both, the beach and the cliff. Furthermore, the iPhone’s orientation and angle do not need to be regarded during scanning. Nevertheless, differences in the M3C2 distances between beach and cliff face can be seen in the point clouds at Roneklint cliff. Those differences may results from a registration bias coming from the GCPs that were distributed along the cliff. Two other possible reasons could be the different roughness between beach and cliff face or the inaccuracies in the iPhone’s IMU resulting in deficient slope angle projections.

Applications like ‘EveryPoint’ show the future potential for the combination of SfM MVS smartphone photogrammetry and LiDAR. SfM MVS techniques, with images captured by the iPhones cameras, and LiDAR can increase the level of detail in the models as well as the range of the models. However, the handling of the app still requires elaborate data acquisition for the SfM MVS photos e.g. overlap between images, viewing angle, sunlight exposure.

Overall, the LiDAR sensor introduced by Apple Inc. in 2020 for the iPad Pro and iPhone Pro models presents a novel, cost effective and time efficient alternative to established methods of topographic land surveying like TLS and SfM MVS that is capable of rapidly scanning the topography of small to medium scale landforms in high spatial resolution. Although accuracy and precision of the iPhone LiDAR models do not reach state-of-the-art SfM MVS standards hitherto, the LiDAR sensor is capable of realistically representing environments like the coastal cliff of Roneklint above a threshold of 10 cm. Improvements in raw point cloud export, maximum scan size and range are only a matter of time for the still novel software applications. With the iPhone’s LiDAR primary field of application in small-scale landforms it offers advantages in accessibility, usability and integrated data processing.

## Methods

The devices tested in this study are the Apple iPad Pro 2020 12.9-inch display (iPad), 2020 and the Apple iPhone 12 Pro (iPhone).

### Technical capabilities

To test accuracy of the LiDAR sensor, 14 rectangular boxes with sharp edges were scanned and the dimensions were measured with a measuring stick. The dimensions of the objects scanned range from 14 × 6 × 2 cm up to 50 × 30 × 52 cm. Five boxes were scanned 5–7 times to account for precision of the scans (Fig. 3, Supplementary Table S2). The point density and capturing mode of the LiDAR sensor were measured by centering the iPad and the iPhone in front of a 20 × 20 cm flat white square with distances ranging from 25 to 250 cm at intervals of 10 to 25 cm (Supplementary Table S1). Points within the square were counted on photos taken with a Raspberry Pi Camera Board NoIR v2.1 (8 MP), that comes without an infrared filter making the laser dots visible (Supplementary Figs. S3 and S4).

### Reference point clouds

Reference point clouds were created using a SfM MVS method^32^. Photos for SfM were taken with a DJI Matrice 210 RTK UAV and the X5S Zenmuse camera system in December 2020 with a resolution of 5280 × 3956 pixels and with the iPhone wide camera in September 2021 with a resolution of 4032 × 3024 pixels. 20 GCPs were distributed along the cliff at Roneklint for co-registration of point clouds. SfM and MVS were performed in AgiSoft Metashape Professional, version 1.6.5. For the first reference point cloud of Roneklint 138 photos were taken with the DJI Matrice 210 RTK UAV in December 2020, creating a point cloud with 235,647,817 points (Supplementary Fig. S5). The SfM MVS point cloud was scaled utilizing the local RTK reference system of the UAV. In September 2021 photos of a 15 × 10 × 10 m area at the cliff of Roneklint were taken with the iPhone 12 Pro wide camera two times. Two SfM MVS point clouds were created with 1,017,016 points and 888,519 points. A statistical outlier removal filter was applied to all SfM MVS point clouds and the point clouds were subsampled with a minimum spacing of 0.01 m between points in CloudCompare^25^.

### LiDAR scanning

Several Apps are available for creating a 3D model of a surface with the iPad and iPhone LiDAR sensor (e.g. ‘3d Scanner App’, ‘EveryPoint’, ‘Polycam’). We used the ‘3d Scanner App’ version 1.8.1 by Laan Labs in December 2020 and version 1.9.3 in September 2021. In between the scanning dates, GNSS tagging was added to the app’s functionalities. Point cloud export did not function properly in December 2020 yet, probably due to the large data size. Subsequently, export in December 2020 was done as a mesh in the OBJ format, whereas the LiDAR scans in September 2021 were exported as point clouds in the LAS format.

When using the above-mentioned applications, a mesh is compiled on the go with the build-in three-axis gyroscope working as an inertial measurement unit. Apple Inc. proprietary software platform ARKit triangulates the mesh internally based on the raw point measurements. During point cloud export with the ‘3d Scanner App’ points are sampled from the mesh’s surface and the points are not the raw point cloud collected with the iPhone’s LiDAR sensor. Apps like ‘SiteScape’ and ‘EveryPoint’s’ ARKit LiDAR Points scanning mode allow direct point cloud recording and export, but the applications are limited to a maximum of 12 million points at the moment, making them unsuitable for this study. Using mesh scanning applications allows the scanning of much larger areas and the creation of bigger 3D models.

Scanning with the ‘3d Scanner App’ was conducted by walking along the cliff as well as up and down the beach with the hand-held iPhone or iPad, covering every angle of the object of interest. Furthermore, the iPhone was mounted on a 1.5 m long hand-held selfie stick to extend the area of investigation. Re-scanning within one scan is possible and results in overwriting of previously covered areas. During the scanning, the build-in 12 mega-pixel wide-angle camera is taking additional images that are used to add texture to the scan afterwards (Supplementary Fig. S1).

In December 2020, the entire cliff of Roneklint was scanned with the iPhone 12 Pro, and a 10 × 15 × 10 m area is scanned four times with the iPhone, and two times with an iPad. In September 2021 the latter area was scanned two times with the ‘3d Scanner App’ again and recordings of the cliffs were obtained with the ‘EveryPoint’ app by URC Ventures Inc. version 2.5 in the ARKit LiDAR Mesh mode. In that scanning mode, a mesh of the close surroundings (< 5 m) is generated based on the iPhone’s LiDAR sensor. At the same time, the app is taking a video with the iPhone’s camera. Data recoding is performed by walking along the beach close to the water line pointing the phone side wards of the moving direction towards the object of interest. Close surroundings like the beach are captured by the LiDAR sensor, whereas objects further away like the cliff, are only recorded in the video. Both video and LiDAR mesh were uploaded to EveryPoint’s servers where their own photogrammetry algorithm is creating a point cloud out of stills cropped from the video and the LiDAR mesh. The user can download the point cloud that combines images and LiDAR data as well as the source images when processing is done.

### Point cloud distance calculation

The iPad and iPhone LiDAR data of Roneklint cliff obtained with the ‘3d Scanner App’ in December 2020 and September 2021, as well as the ‘EveryPoint’ point clouds were exported and loaded into CloudCompare^25^. The point clouds were co-registered to the SfM MVS reference point clouds based on the GCPs distributed throughout the study area. During initial point cloud alignment and fine registration the LiDAR and ‘EveryPoint’ point clouds were not scaled to the SfM MVS point clouds, and the original dimensions were maintained. To harmonize point density, all point clouds were sub-sampled with a minimum distance of 0.01 m between points (Fig. 1).

Distances between SfM MVS, ‘EveryPoint’ and LiDAR based point clouds are calculated with a multi-scale model-to-model cloud comparison (M3C2) approach^26^. The M3C2 distance calculation is solely based on point cloud comparison and is therefore chosen over a method interpolating surfaces (e.g. Cloud to Model & Digital Elevation Model of Difference) as M3C2 is more reliable on complex topographies^33^. Furthermore, the M3C2 approach is well adapted to calculate distances between two point clouds on a cliff, as it is suitable for vertical as well as horizontal surfaces and it gives positive and negative values of distance^34^. The iPhone LiDAR scan of the entire cliff from December 2020 is compared to the reference SfM MVS cloud. Further three iPhone and two iPad scans of a smaller part of the cliff are compared with one iPhone scan as a reference scan to test model-to-model precision on a stable target in a real world environment. The two iPhone camera SfM MVS, two ‘3d Scanner App’ LiDAR and two ‘EveryPoint’ models from September 2021 are compared to each other with one of the SfM MVS point clouds as a reference. The M3C2 distance calculations are uniformly executed at 0.01 m minimal nominal spacing with normal directions and projections diameters calculated at 1.33 m for each point at the entire cliff and 0.15 m at the smaller part.

## Supplementary Information

Supplementary Information.

## Data Availability

Data is available under the following link: https://doi.org/10.6084/m9.figshare.13382750.
