# Supplementary material for: Evaluation of the Apple iPhone 12 Pro LiDAR for an Application in Geosciences
Source: Sci Rep. 2021 Nov 15;11:22221. doi: 10.1038/s41598-021-01763-9 (PMC8593014; doi:10.1038/s41598-021-01763-9)
Supplement: Supplementary file 1 — Supplementary Information. [file 41598_2021_1763_MOESM1_ESM.docx]

*Scientific Reports*

Supporting Information for

**Evaluation of the Apple iPhone 12 Pro LiDAR for an Application in Geosciences**

G. Luetzenburg, A. Kroon, and A. A. Bjørk

Department of Geosciences and Natural Resource Management, University of Copenhagen

**Contents of this file**

Figures S1 to S18

Tables S1 to S3

**Introduction**

This SI provides additional information about the method to calculate the point density of the iPad and iPhone LiDAR sensor (Figure S1-S4, Table S1-S2), the SfM based reference point cloud (Figure S5), the M3C2 distance calculations of the entire cliff of Roneklint (Figure S6-S8) and the M3C2 distance calculations of a small area of the cliff (Figure S9-S19).

Data for point density, precision and accuracy was acquired in November 2020. Photos for the SfM MVS point cloud and the iPad and iPhone meshes were captured on 08 December 2020 and 07 September 2021. Data processing was carried out after December 08 2020. The steps used for data processing are described in Figure 1 of the main publication.

It was not possible to acquire accurate dGPS data for the GCPs distributed along the cliff. Therefore, the SfM point cloud is scaled locally with the build-in RTK system of the UAV and the point clouds are referenced to each other and to the RTK system, but not accurately globally.


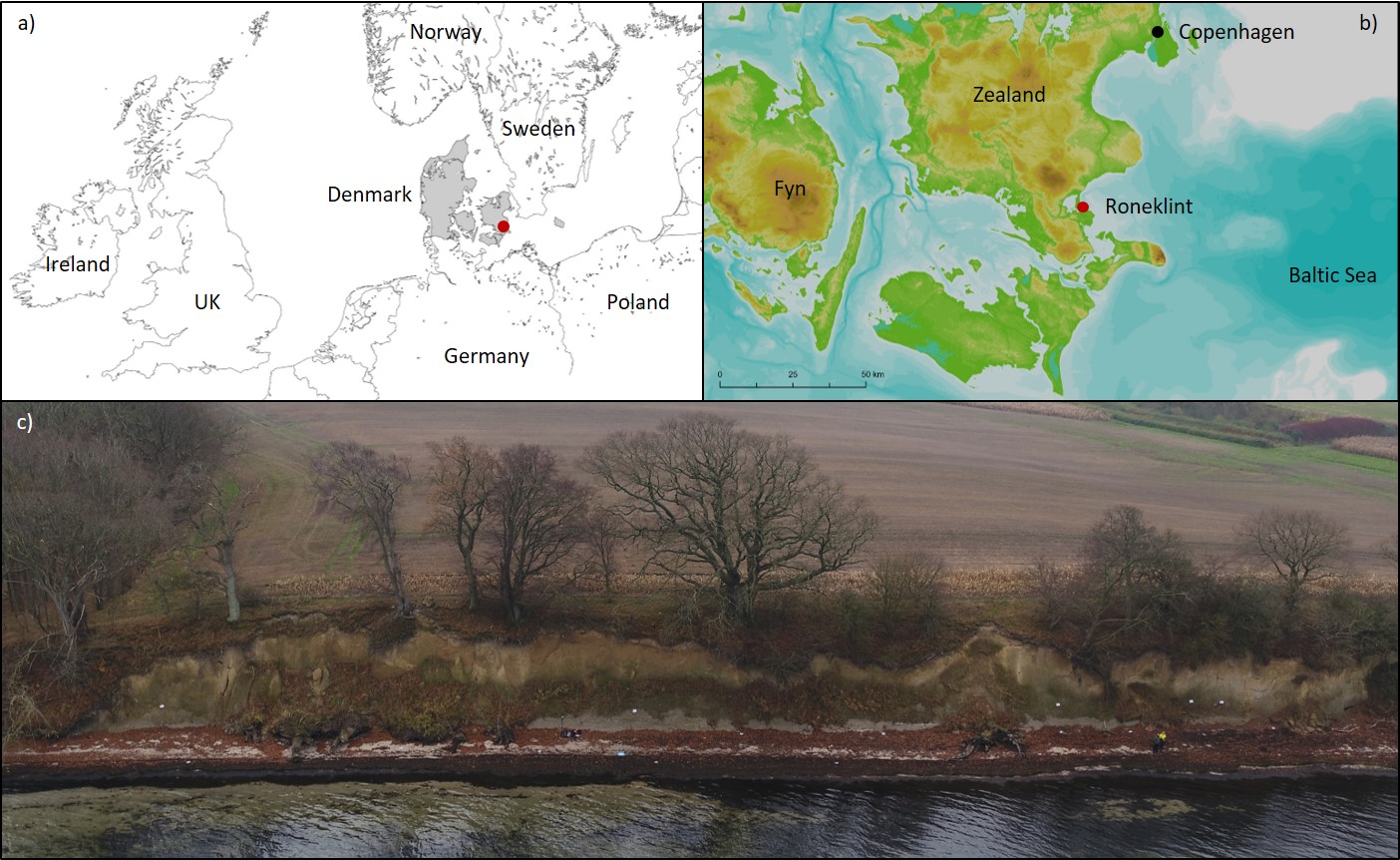


Figure S1. Fig. 1: Northern Europe with the location of the coastal cliff of Roneklint (a), Islands of eastern Denmark with the location of Roneklint facing northeast to the Baltic Sea (b), Aerial image of the 130 m wide coastal cliff of Roneklint (c). Fig. S1 a&b were generated by ArcGIS Pro that was obtained from https://www.esri.com/en-us/arcgis/products/arcgis-pro/, and the topographic data used in Fig. S1b was downloaded from the Geological Survey of Denmark and Greenland (https://eng.geus.dk/products-services-facilities/data-and-maps/maps-of-denmark)


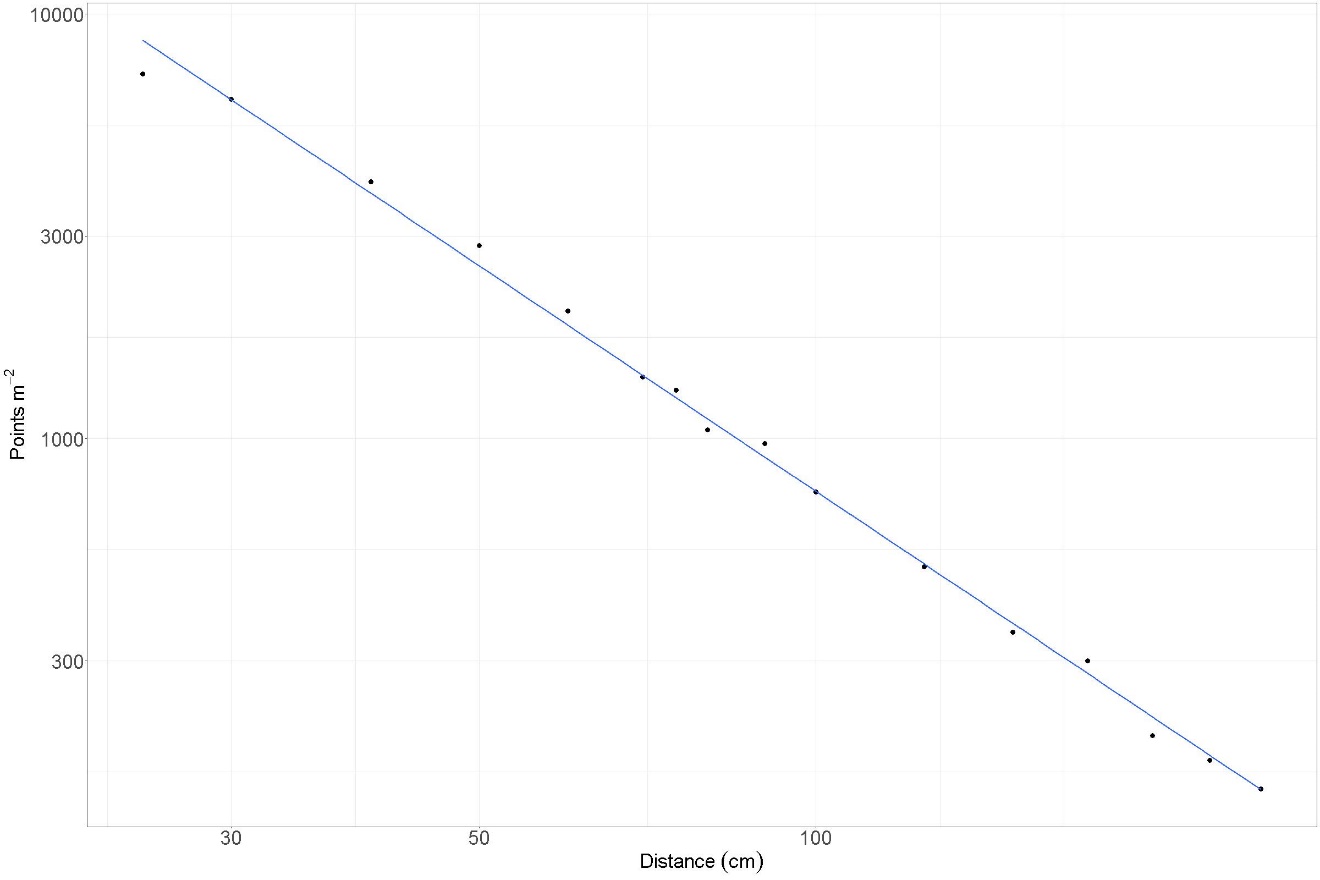


Figure S2. Theoretical point density per square meter between 25 and 250 cm distance to an object following a linear trend on a logarithmic scale.


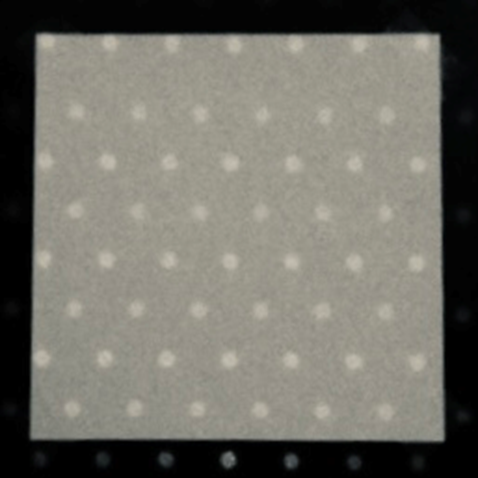


Figure S3. Photo taken with a Raspberry Pi Camera Board NoIR v2.1, showing 52 dots within a 20x20 cm target with a distance of 75 cm to the LiDAR sensor.


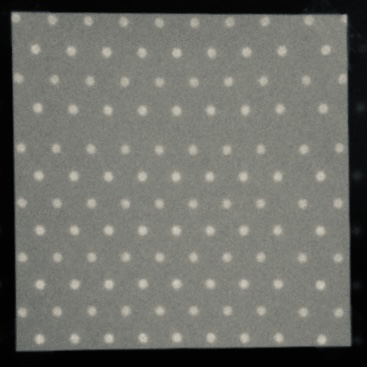


Figure S4. Photo taken with a Raspberry Pi Camera Board NoIR v2.1, showing 114 dots within a 20x20 cm target with a distance of 50 cm to the LiDAR sensor.


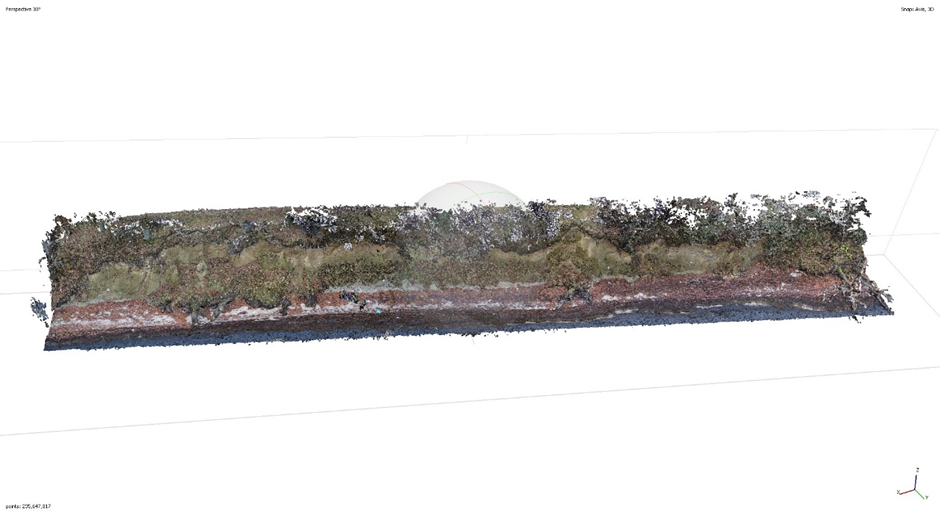


Figure S5. SfM reference point cloud of the entire cliff of Roneklint, based on 138 photos, captured with a DJI Matrice 210 RTK and the X5S Zenmuse camera system with a resolution of 5280x3956 pixels per photo.


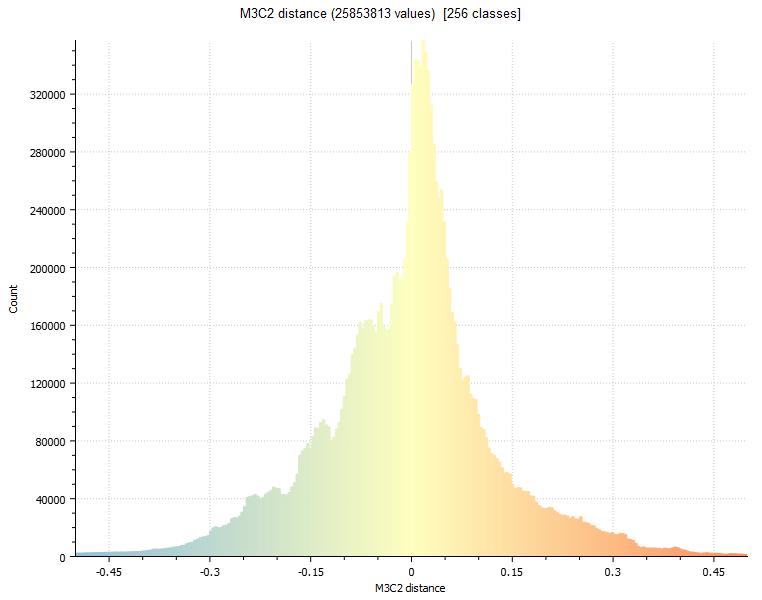


a)

b)


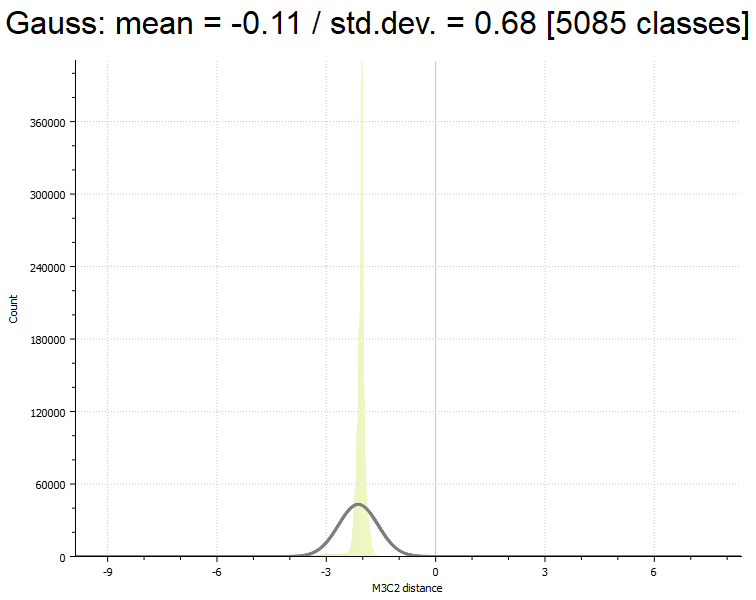


Figure S6. M3C2 distances histogram (m) between reference SfM cloud and iPhone point cloud of the entire cliff of Roneklint (a) Gauss distribution with mean = -0.11, std. dev. = 0.68, RMS = 0.68 (b)


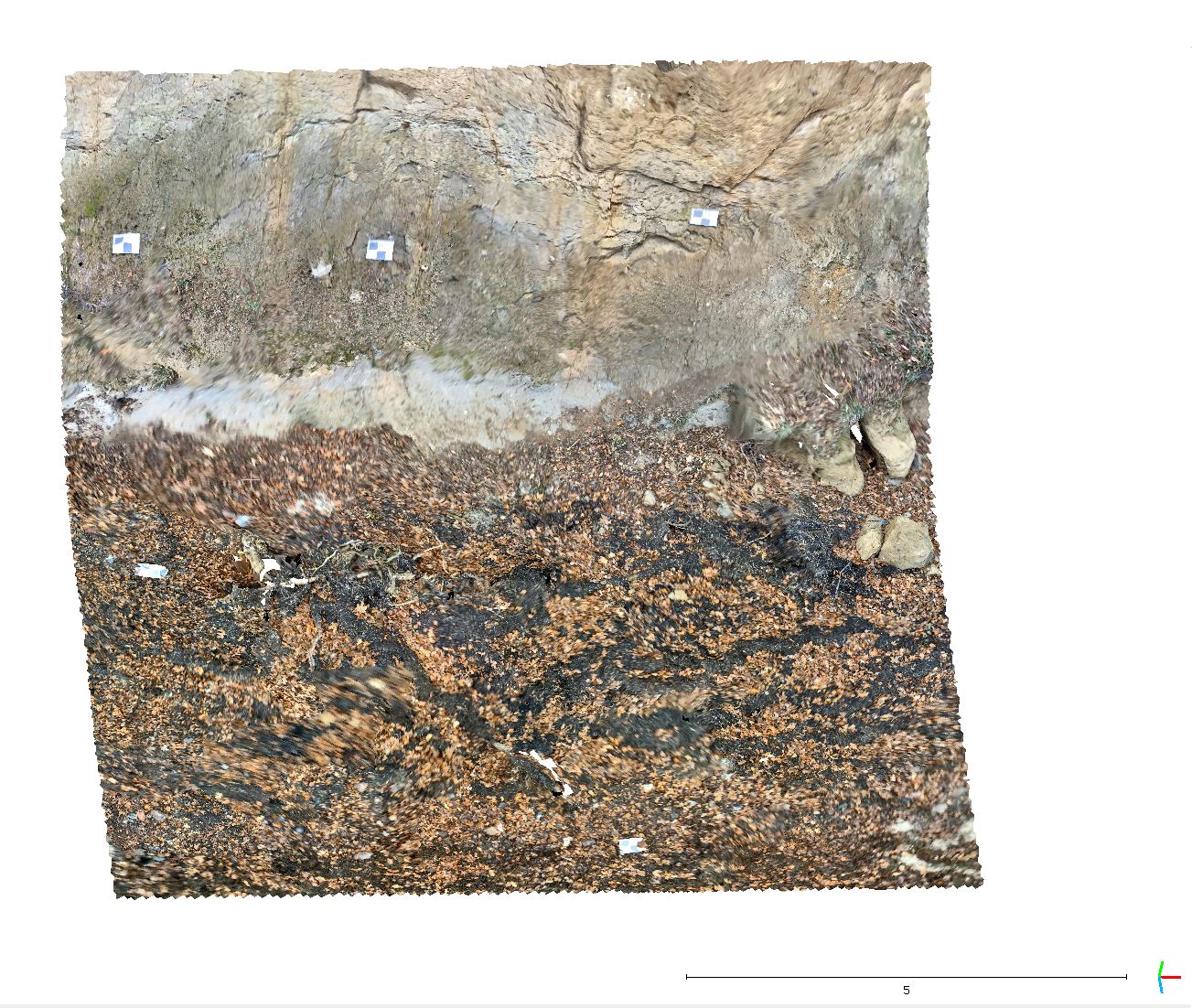


Figure S7. Textured iPhone LiDAR mesh of an area with the dimensions of 10 x 15 x 10 m at the cliff with ground control points on the cliff face and on the beach in front of the cliff. The first out of six scans is used as a reference scan.


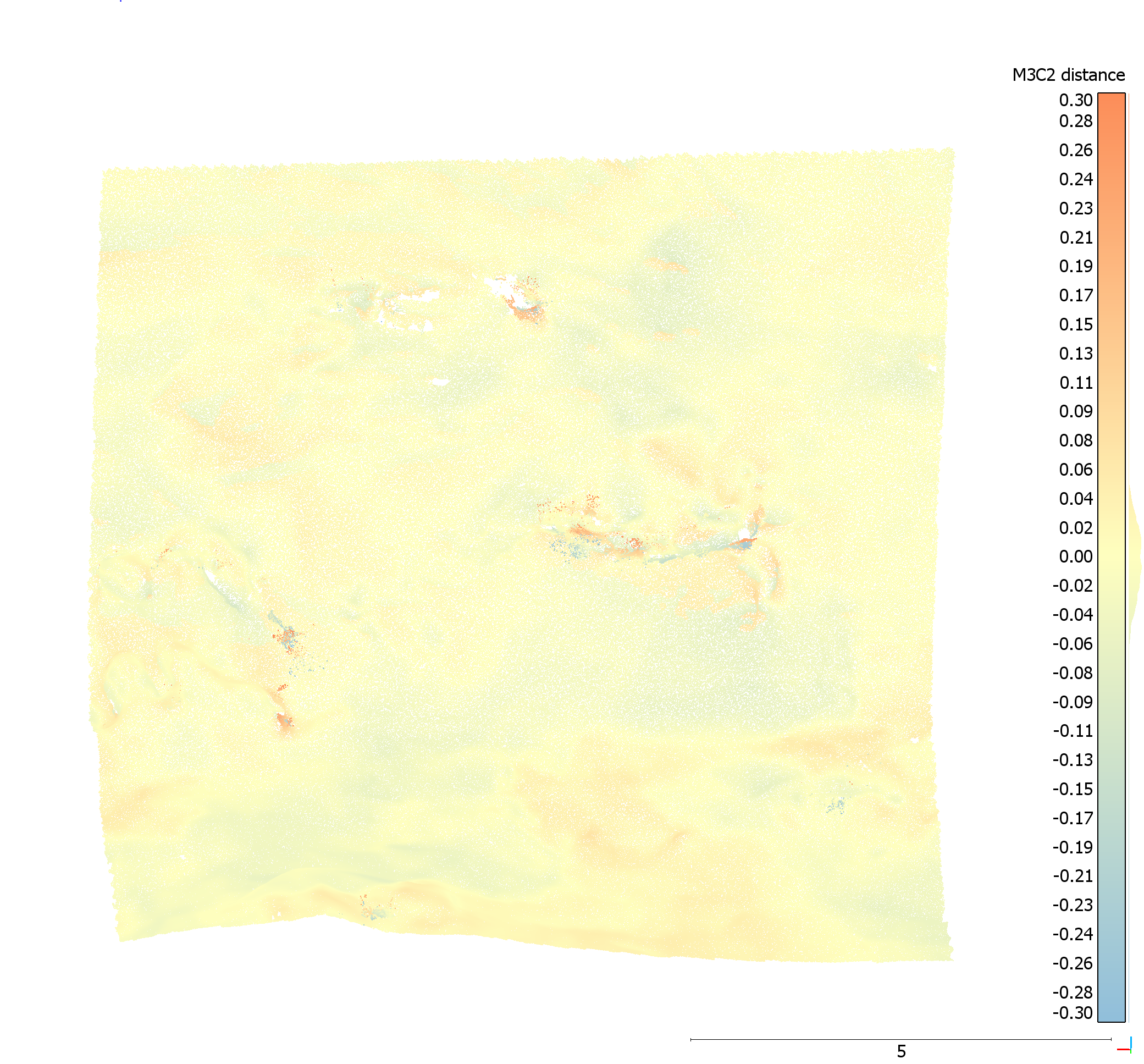


Figure S8. M3C2 distances (m) between iPhone LiDAR reference cloud and iPad scan 01, fine registration error RMS: 0.0381185 m computed on 39,907 points with a theoretical overlap: 80%, point clouds subsampled to 0.01 m minimal nominal spacing between points with normal directions and projections diameter calculated at 0.15 m for each point.


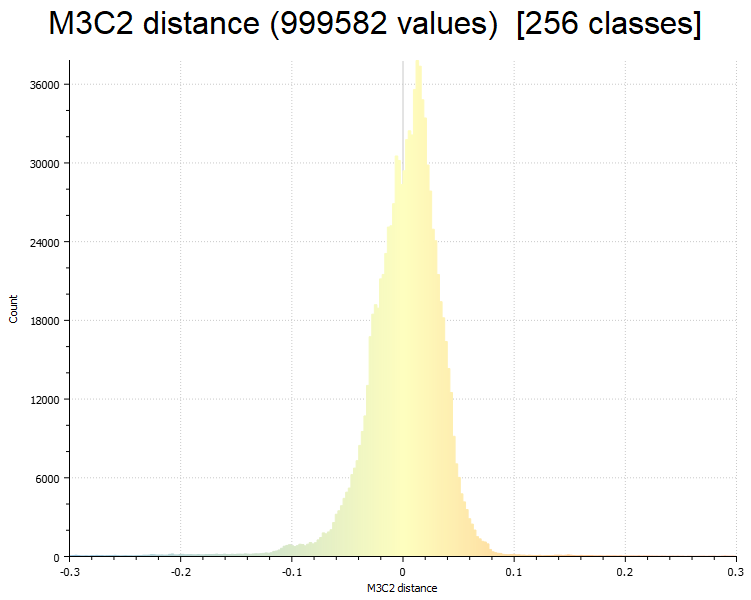


a)

b)


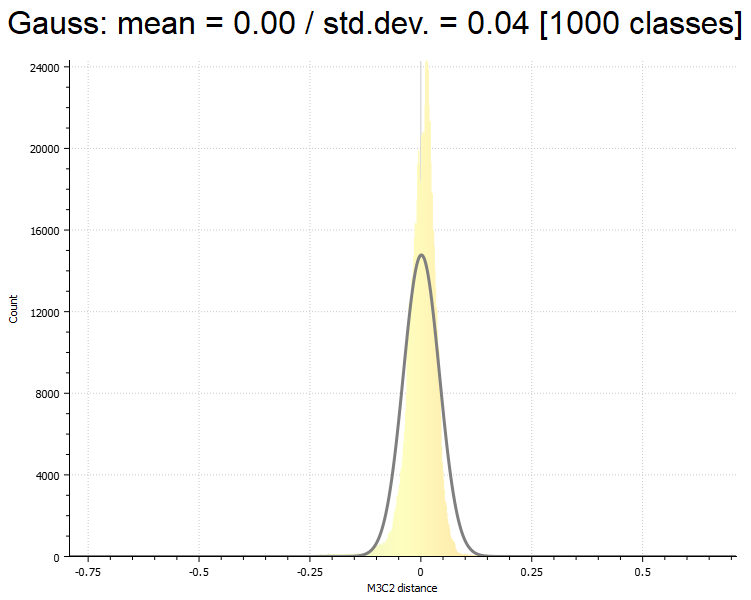


Figure S9. M3C2 distances histogram (m) between reference cloud and iPad scan 01 (a) Gauss distribution with mean = -0.00, std. dev. = 0.04, RMS = 0.04 (b)


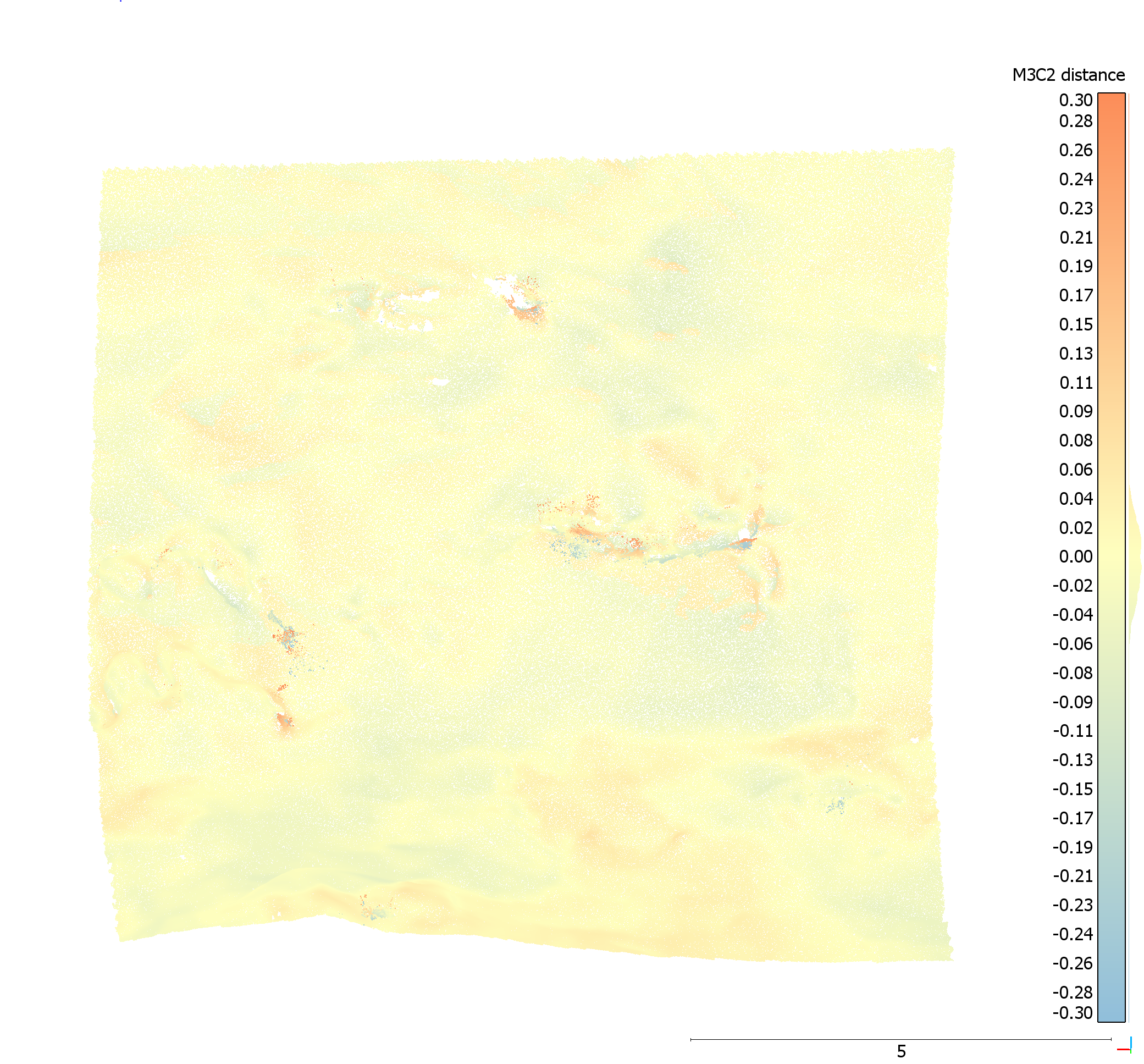


Figure S10. M3C2 distances (m) between the iPhone LiDAR reference cloud and small iPad scan 02, fine registration error RMS: 0.0223082 m computed on 44,980 points with a theoretical overlap: 90%, point clouds subsampled to 0.01 m minimal nominal spacing between points with normal directions and projections diameter calculated at 0.15 m for each point.


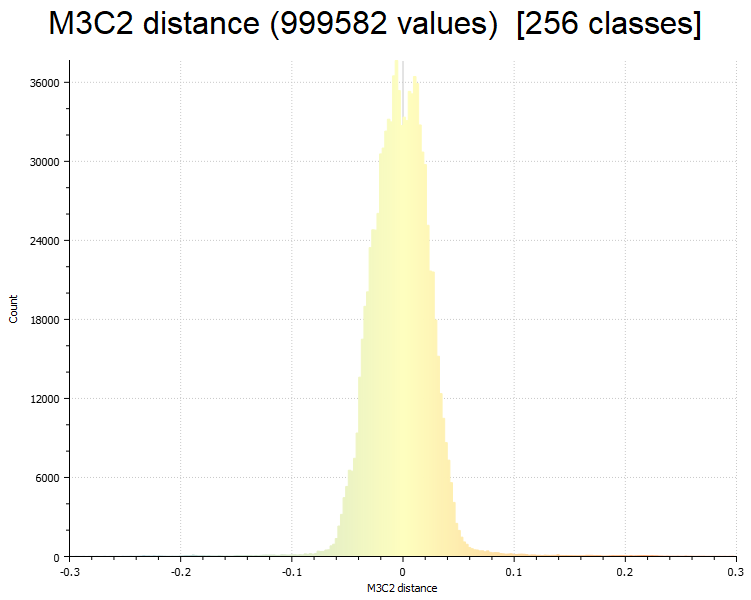


a)

b)


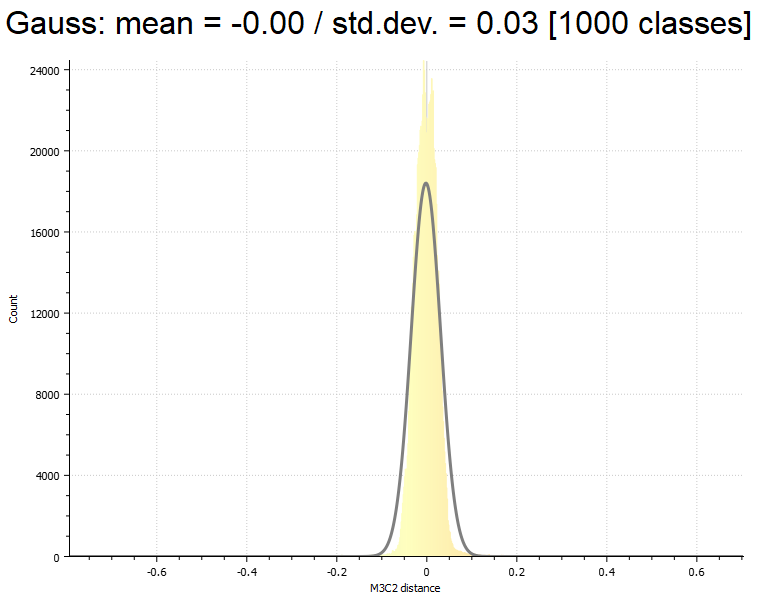


Figure S11. M3C2 distances histogram (m) between reference cloud and iPad scan 02 (a) Gauss distribution with mean = -0.00, std. dev. = 0.03, RMS = 0.03 (b)


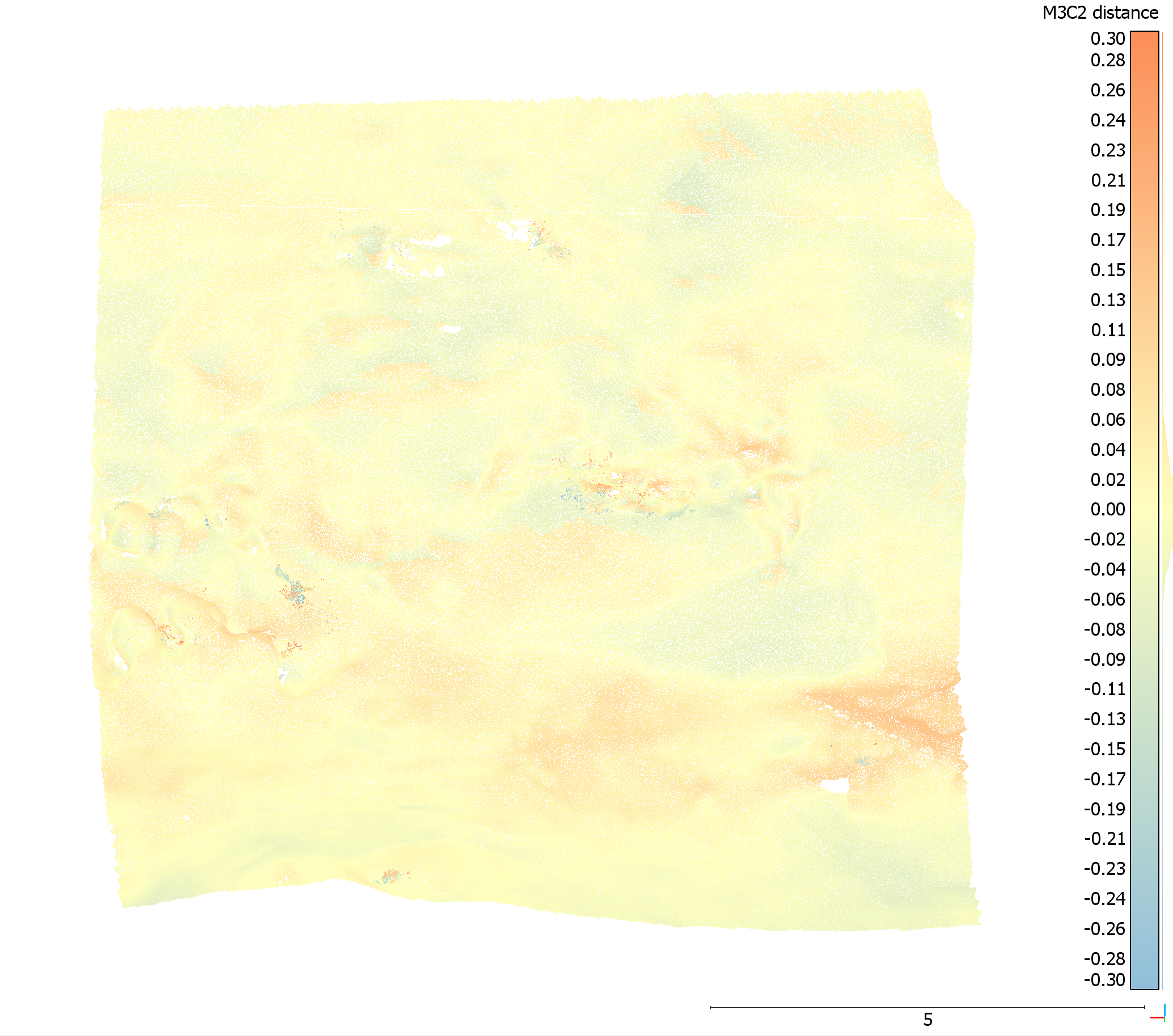


Figure S12. M3C2 distances (m) between the iPhone LiDAR reference cloud and small iPhone scan 02, fine registration error RMS: 0.0391387 m computed on 44,702 points with a theoretical overlap: 90%, point clouds subsampled to 0.01 m minimal nominal spacing between points with normal directions and projections diameter calculated at 0.15 m for each point.


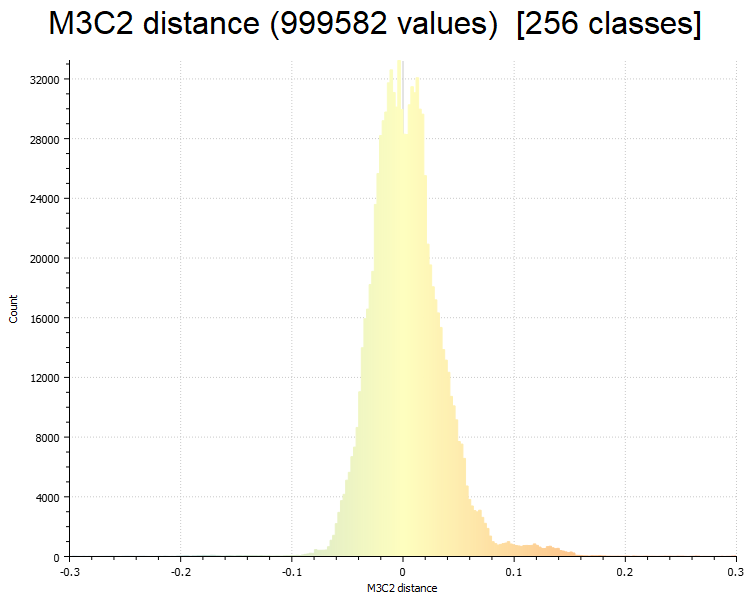


a)

b)


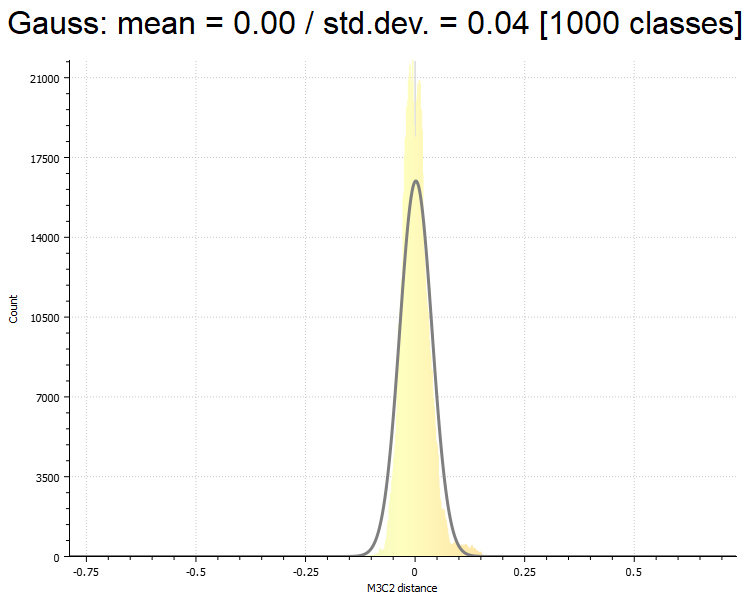


Figure S313. M3C2 distances histogram (m) between reference cloud and iPhone scan 02 (a) Gauss distribution with mean = -0.00, std. dev. = 0.04, RMS = 0.04 (b)


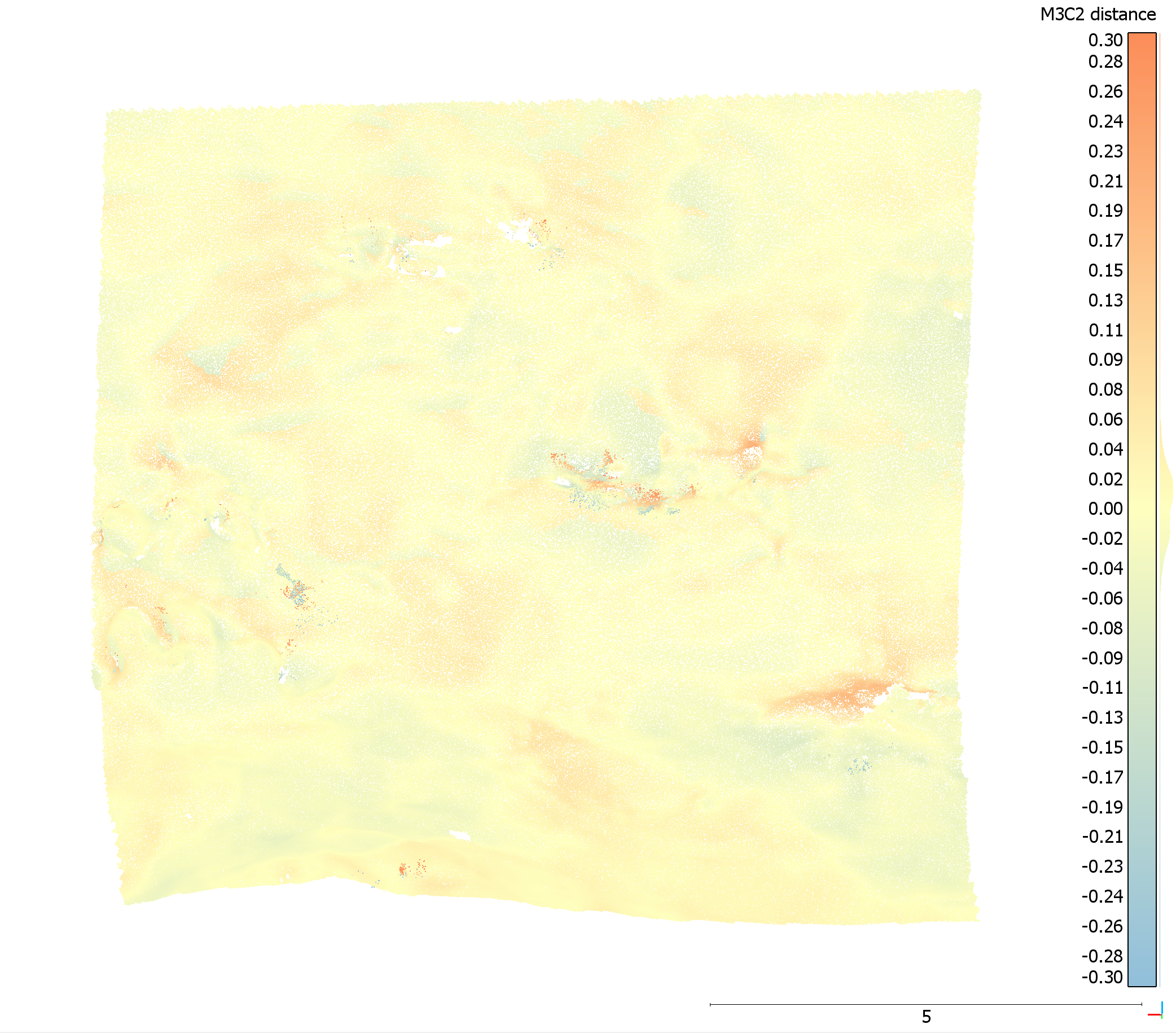


Figure S14. M3C2 distances (m) between the iPhone LiDAR reference cloud and small iPhone scan 03, fine registration error RMS: 0.0267714 m computed on 40,144 points with a theoretical overlap: 80%, point clouds subsampled to 0.01 m minimal nominal spacing between points with normal directions and projections diameter calculated at 0.15 m for each point.


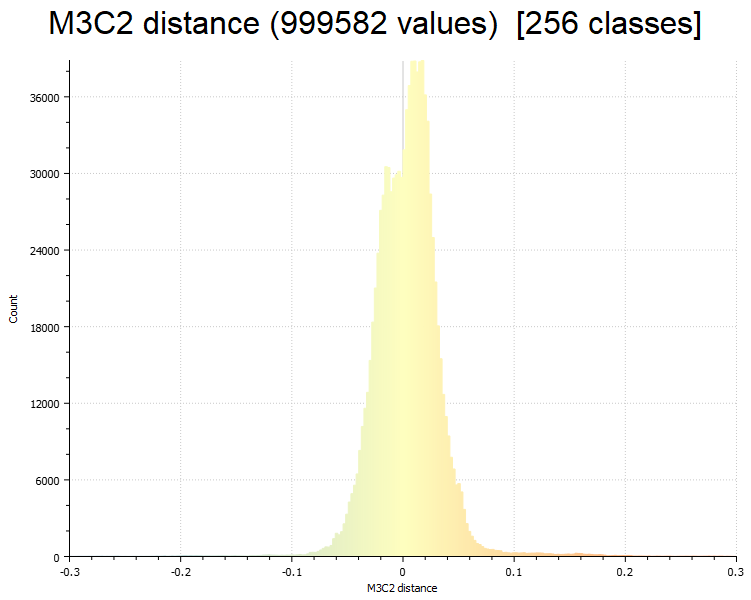


a)

b)


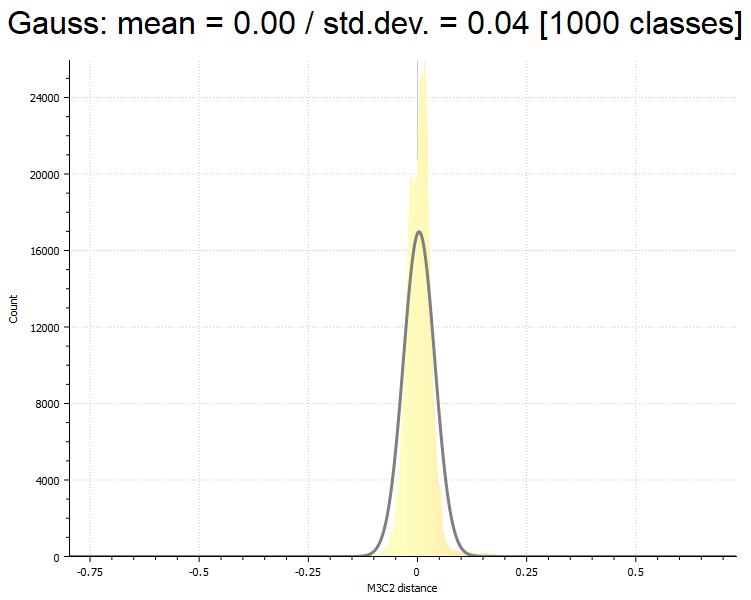


Figure S15. M3C2 distances histogram (m) between reference cloud and iPhone scan 03 (a) Gauss distribution with mean = -0.00, std. dev. = 0.04, RMS = 0.04 (b)


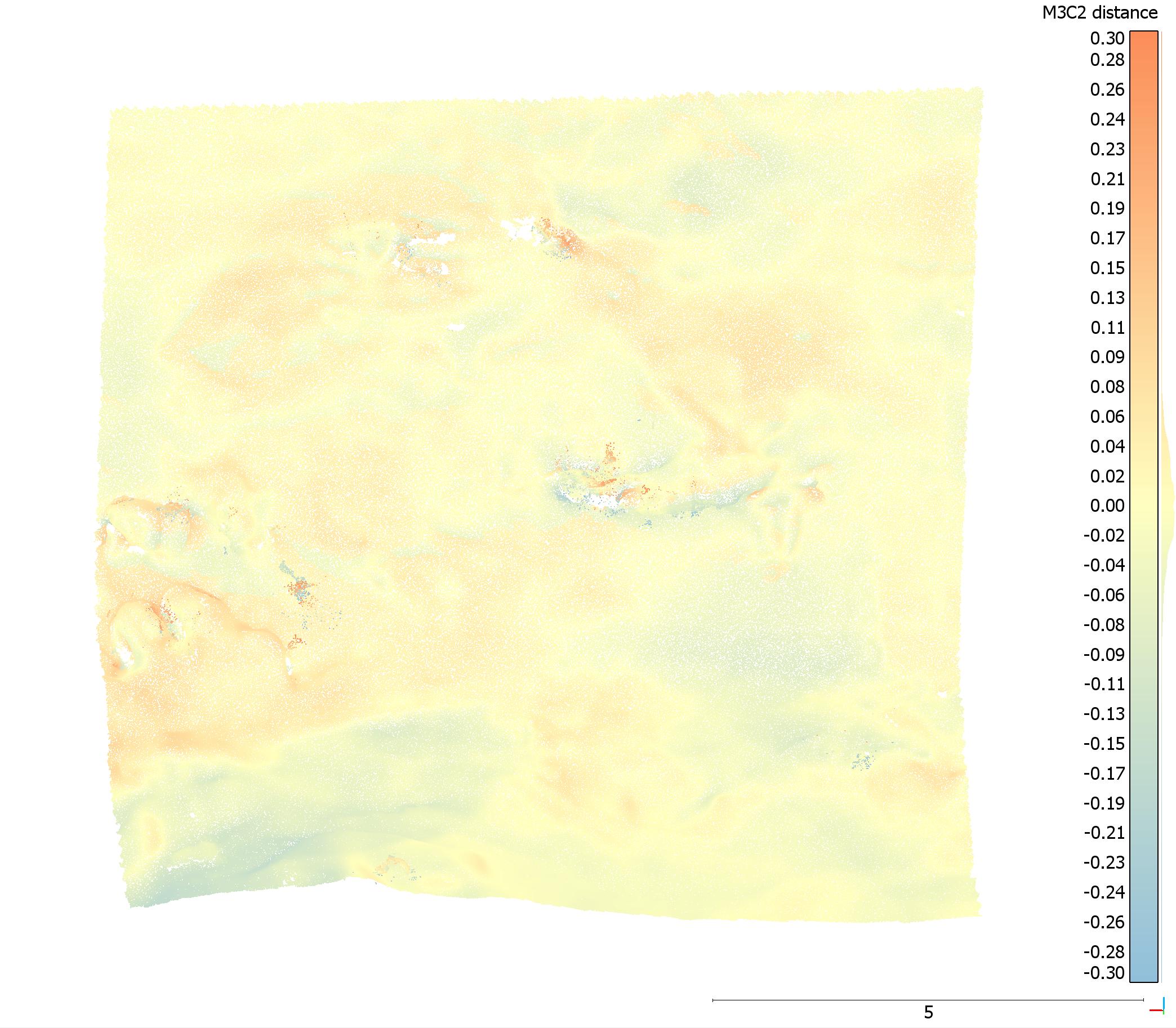


Figure S16. M3C2 distances (m) between the iPhone LiDAR reference cloud and small iPhone scan 04, fine registration error RMS: 0.0752684 m computed on 39,827 points with a theoretical overlap: 80%, point clouds subsampled to 0.01 m minimal nominal spacing between points with normal directions and projections diameter calculated at 0.15 m for each point.


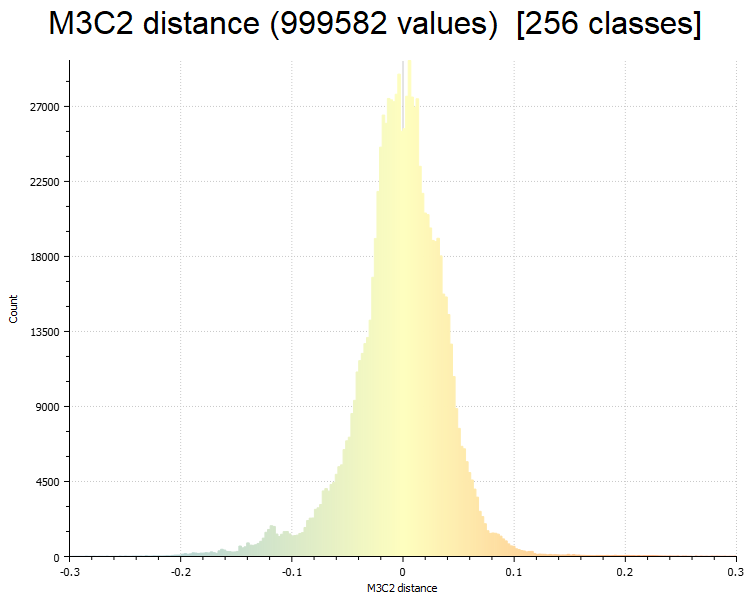


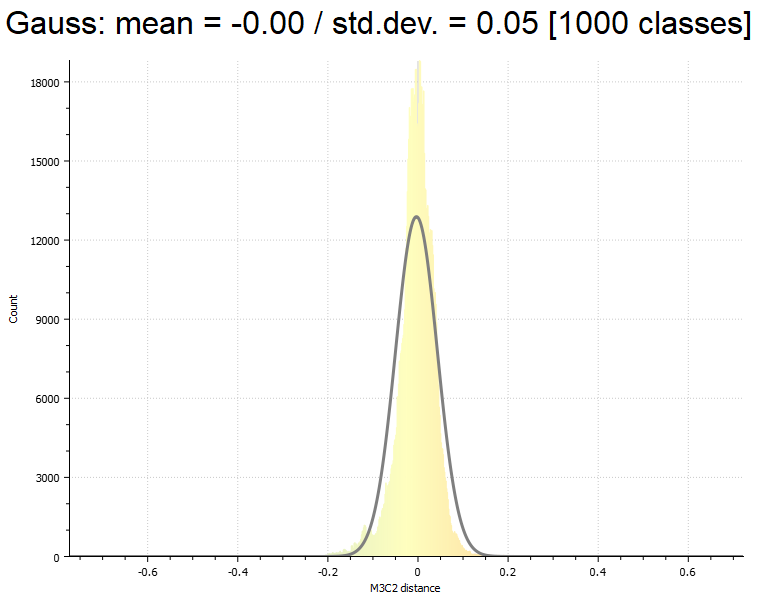


**Figure S17.** M3C2 distances histogram (m) between reference cloud and iPhone scan 04 (a) Gauss distribution with mean = -0.00, std. dev. = 0.05, RMS = 0.05 (b)

**
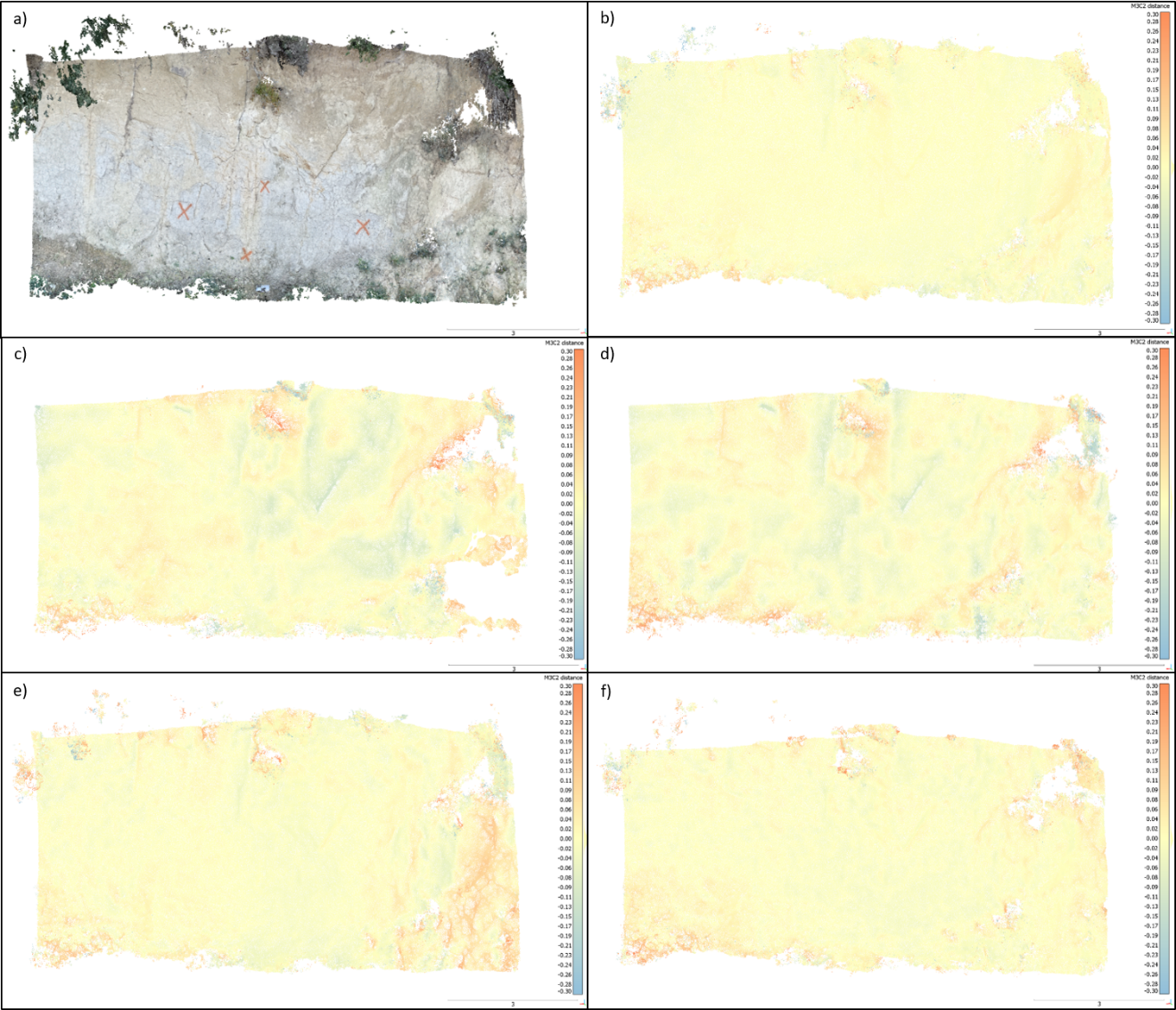
**

Figure S18. M3C2 distances from September 2021 of a bare soil cliff face area at Roneklint with the iPhone camera SfM MVS reference cloud (a), the M3C2 iPhone camera SfM MVS cloud 02 (b) the M3C2 ‘3d Scanner App’ 01 (c), the M3C2 ‘3d Scanner App’ 02 (d), the M3C2 ‘EveryPoint’ 01 (e) and the M3C2 ‘EveryPoint’ 02 (f) point clouds.

| Distance to target (cm) | Number of points within target (20x20 cm) | Extrapolated points per square meter (x25) |
| --- | --- | --- |
| 25 | 289 | 7225 |
| 30 | 252 | 6300 |
| 40 | 161 | 4025 |
| 50 | 114 | 2850 |
| 60 | 80 | 2000 |
| 70 | 56 | 1400 |
| 75 | 52 | 1300 |
| 80 | 42 | 1050 |
| 90 | 39 | 975 |
| 100 | 30 | 750 |
| 125 | 20 | 500 |
| 150 | 14 | 350 |
| 175 | 12 | 300 |
| 200 | 8 | 200 |
| 225 | 7 | 175 |
| 250 | 6 | 150 |

Table S1. Measured points emitted from the LiDAR scanner within a 20x20 cm square at distances from 25 to 250 cm.

| No | Side | Size (cm) | Similarity (%) | Standard deviation |
| --- | --- | --- | --- | --- |
| H1 | length | 23 | 98,18 | 2,49 |
| H2 | length | 38 | 97,89 | 1,18 |
| H3 | length | 22 | 97,39 | 2,38 |
| H4 | length | 13 | 94 | 5,85 |
| O1 | length | 16 | 85,71 | NA |
| O2 | length | 20 | 100 | NA |
| O3 | length | 22 | 95,65 | NA |
| O4 | length | 40 | 100 | NA |
| O5 | length | 49 | 98 | NA |
| O6 | length | 40 | 95,24 | NA |
| O7 | length | 30 | 96,77 | NA |
| O8 | length | 12 | 92,31 | NA |
| O9 | length | 10 | 83,33 | NA |
| H1 | width | 23 | 97,27 | 2,49 |
| H2 | width | 29 | 98,57 | 1,96 |
| H3 | width | 13 | 94,82 | 8,19 |
| H4 | width | 13 | 89,24 | 11,3 |
| O1 | width | 9 | 50 | NA |
| O2 | width | 10 | 90,91 | NA |
| O3 | width | 16 | 94,12 | NA |
| O4 | width | 31 | 100 | NA |
| O5 | width | 29 | 96,67 | NA |
| O6 | width | 36 | 100 | NA |
| O7 | width | 11 | 91,67 | NA |
| O8 | width | 8 | 88,89 | NA |
| O9 | width | 9 | 87,5 | NA |
| H1 | height | 10 | 97,78 | 4,97 |
| H2 | height | 37 | 98,95 | 1,44 |
| H3 | height | 6 | 79,14 | 18,23 |
| H4 | height | 4 | 85,33 | 14,45 |
| O1 | height | 3 | 50 | NA |
| O2 | height | 5 | 100 | NA |
| O3 | height | 6 | 100 | NA |
| O4 | height | 12 | 100 | NA |
| O5 | height | 52 | 100 | NA |
| O6 | height | 41 | 94,87 | NA |
| O7 | height | 9 | 87,5 | NA |
| O8 | height | 8 | 100 | NA |
| O9 | height | 8 | 40 | NA |

Table S2. Measured values from the scanned meshes (Size) and the similarity to the real values (Similarity) to test accuracy and precision of the LiDAR scanner. The values are obtained by scanning rectangular boxes with sharp edges with the ‘3d Scanner App’ and measuring the dimensions with a measuring stick.

| **M3C2 point cloud** | **Mean Gauss distribution** | **Std. dev.** | **RMS** |
| --- | --- | --- | --- |
| iPhone camera SfM MVS 02 | 0.00 | 0.05 | 0.05 |
| 3d Scanner App 01 | 0.00 | 0.08 | 0.07 |
| 3d Scanner App 02 | 0.00 | 0.08 | 0.08 |
| EveryPoint 01 | 0.01 | 0.07 | 0.07 |
| EveryPoint 02 | 0.01 | 0.07 | 0.07 |

**Table S3.** Average M3C2 distances in meter between the iPhone camera SfM MVS reference cloud and iPhone camera, ‘3d Scanner App’ LiDAR and ‘EveryPoint’ photogrammetry + LiDAR point clouds from a bare soil cliff area at Roneklint measured in September 2021.
